# COVID-19 and heterogeneous vulnerabilities in the Peruvian labor market: implications for social inequalities and for gender gaps

**DOI:** 10.1007/s40888-021-00245-5

**Published:** 2021-09-23

**Authors:** Rosa Luz Durán

**Affiliations:** Department of Economics, Universidad de Lima, Lima, Peru

**Keywords:** COVID-19, Gender gap, Employment, Job loss, Peru, I14, I38, J21, J22

## Abstract

Using quarterly data from the 2020 Peruvian National Household Survey (ENAHO), this paper estimates the differentiated impacts of the COVID-19 pandemic on a set of labor market indicators, such as labor participation, occupational categories, informality, and number of hours worked. The impacts are calculated from an individual perspective (effects on the activities of the heads of household and their spouses, distinguishing them according to sex) and also from a joint strategy perspective among the partners. The results indicate that the intersectionalities of vulnerability considered (rural/urban area, and those contained in the type of households and in the situation of single-parenting or two-parenting of household heads and their spouses) determine that women, who live in rural areas, have children and do not have a partner were the most affected by the global health crisis.

## Introduction

Worldwide, Peru is one of the countries that have been most affected by the COVID-19 crisis. Official information from the Peruvian Ministry of Health (MINSA) reports that as of 21st July 2021, the total number of deaths from COVID-19 was 195,243.^1^ According to the Coronavirus Resource Center at John Hopkins University, by the end of June 2021, Peru was the country with the highest number of deaths per 100 K population in the world (587.21) and the second country with highest case fatality (9.4%).^2^

The pandemic struck the first week of March 2020 when the first case was identified, and on March 15th President Vizcarra officially declared a country-wide lockdown to start the next day, closing national borders, limiting domestic travel, forbidding all business activities deemed non-essential, and severely restricting people’s mobility. Only core activities related to health (functioning of hospitals and pharmacies, being able to care for and move vulnerable family members if needed, with special permits required), food (only buying and selling of staple foods at small local vendors), fuel (production, transportation, distribution and sale), and financial institutions were allowed. The lockdown continued until June 30th 2020, although in May a gradual reactivation of economic activities began, starting with the least risky ones. According to the Stringency Index built by the University of Oxford, which records the strictness of isolation and quarantine policies on a scale from 1 to 100, during these months Peru ranked highest in Latin America, and among the highest in the world.^3^ The drastic social immobilization measures adopted by the Peruvian government caused a collapse in the national gross domestic product, which plunged 30.2% from a year earlier, the deepest slump of any major economy according to Bloomberg (Quigley, 2020).^4^ The coronavirus crisis and the country's entry into a state of national emergency and quarantine had a massive impact on the Peruvian labor market. According to the administrative records of the Ministry of Labor and Employment Promotion (MTPE)’s electronic worksheet and formal employment monitoring board, in March 2020 there was a net loss of 151,099 jobs nationwide, and 160,090 in April of the same year. These private formal sector workers were found in the commerce, services, manufacturing, and construction sectors. In May 2020, the net loss of jobs was lower (39,978). It was only in June that a net increase was registered, which in the third quarter of the year stabilized at a monthly national average increase of 92,000 jobs in the same economic sectors mentioned (MTPE, 2020).

The study of the distributive impact of the pandemic shock is gaining importance, as it becomes increasingly clear that the shock interacts with pre-existing heterogeneities in asset holding, in the ability to generate income, in the access to public services, as well as many others. Growing evidence around the world indicates that women are suffering disproportionately (relative to their male counterparts) from the effects of the crisis triggered by the spread of COVID-19; women are overrepresented in independent and informal economic sectors as well as business and service activities, which have so far been hit the hardest by the pandemic. The lower rates of female employment, and their predominance in part-time jobs, also place women in a particularly fragile situation. In general, the population in informal or low-skilled markets, in precarious employment conditions, historically excluded groups such as those of indigenous descent, and other vulnerable groups are the most exposed to the effects of COVID-19, making them poorer than they already were, with inequality becoming exacerbated (Lustig and Tommasi, 2020).

Using nationally representative quarterly data from the 2020 Peruvian National Household Survey (ENAHO), this paper identifies the most important heterogeneities in the vulnerabilities of different groups of individuals and 'types' of households in the face of the pandemic, and quantifies the impacts on labor participation, occupational category, informality and hours worked of the heads of households and their spouses, both individually and in joint configurations of work activities as a couple. This paper shows how the COVID-19 crisis is affecting workers in various industries, occupations and types of employment in a differentiated way, and what the impacts of this are on inequalities and gender gaps between individuals within the Peruvian labor market. Also considered are the interactions of labor market dynamics with aggravating conditions such as rurality and family composition. An important and distinctive element is the incorporation of an intra-household perspective, which takes into account that workers do not live in isolation, and that their interactions with other workers in the same family, within the home, can alter vulnerabilities to shocks to set in motion strategies for diversifying activities among the members of the couple, for example, that help to better cope with the crisis.

## Literature review

According to United Nations (2020), the International Labor Organization estimates that the total or partial confinement measures have so far affected almost 2.7 billion workers, representing approximately 81% of the world's workforce. Impact studies on households show that a considerable proportion of them disposed of assets or used their savings to cope with the loss of income and to meet regular expenses, or to cover the costs associated with the treatment of the disease (Hevia and Neumeyer 2020; Frisancho et al. 2020; Glover et al. 2020). Other studies, such as that of Mahmud and Riley (2021), highlight the reduction of expenses even in food items, or the reduction in the number of meals per day, as some of the responses of households to this health and economic crisis. Academic studies evaluating the gendered impacts of the pandemic on the labor market and investigating the potential changes in social and economic inequalities resulting from the measures taken by the world's governments to deal with the COVID-19 crisis are still scarce.

Overall, the negative effects of the pandemic on the labor market are more pronounced for female, older, and less-educated workers. In a study for the European Union, Garrote et al. (2020) account for the exposure of this labor market to COVID-19 by identifying jobs most at risk from the pandemic and jobs in non-essential industries that cannot be done from home, and conclude that in high-income European countries, the impact on the labor market is borne disproportionately by young and poorly educated workers, who are employed in low-paying jobs, live in regions that are already lagging behind, and are subject to a higher prevalence of temporary employment contracts. Thus, the crisis over the pandemic is destined to exacerbate inequality, both within and between countries, unless drastic corrective measures are taken. The situation of lower-income countries, such as those in Latin America, is likely to be more difficult, as large portions of the workforce operate in the informal sector and underlying socioeconomic inequalites come into play. Indeed, for Peru, Jaramillo and Ñopo (2020) address the issue of the labor market and women's work in particular and find that gender gaps in employment and in the ability to generate income are accentuated when they interact with worsening factors such as poverty, rurality and ethnicity.  Bargain and Aminjonov (2021) made the  observation that individuals living in poverty cannot really comply with the partial or total quarantine measures determined by their governments since they must continue working in some way to support their families, and evaluated whether mobility for work reasons was related to the local intensity of poverty. Using poverty and mobility data from Google for 242 regions in nine countries in Latin America and Africa, the authors found that the reduction in mobility for work reasons during quarantine periods was indeed lower in high-poverty regions compared with other regions.

In a study of six countries with different geographic locations and different levels of per capita income (China, Italy, Japan, South Korea, the United Kingdom and the United States), Dang and Nguyen (2021) found that while no gender differences were apparent regarding the temporary loss of employment, women were 24% more likely than men to lose their jobs permanently as a result of the pandemic. Factors such as participation rates differentiated by industry between men and women were indicated as the authors as an important part of the explanation of this finding and of other gender gaps, such as those in lost income, expenses and expected savings. Other authors delved further into the role of specific occupations and economic sectors. For instance, Holder  et al. (2020) examined the impact of COVID-19 on the employment of black women in the United States in terms of their occupational and industrial distribution, as well as labor participation and hours of work, finding that although the economic crisis caused by the pandemic has been generalized, it has not been shared equally between races and genders. Black women were disproportionately affected because of their high levels of occupational and industrial segregation in the labor market, and also because they are, along with black men, overrepresented in the room and board industry, where there are notoriously low wages. Thus, both by gender and by race, job losses strongly impacted black women. Unlike in previous economic recessions, the authors argue, government officials ordered industries previously considered immune to recessions, and which happen to have overrepresentation of women, to stop or significantly slow down their activity. This coincides with Alon et al. (2020) finding that the COVID-19 crisis is affecting gender inequality differently than how conventional recessions have affected it in the past, due to the interactions that have been generated among the adoption of flexible jobs, family labor reassignments both inside and outside the home, and the harder restrictions suffered by the activities of economic sectors with high rates of female participation.

Agarwal (2021), in her study on India, examines the dynamics and vulnerabilities within the household and their impact on women as a consequence of job losses in both sexes caused by COVID-19, particularly in poor households. Her main conclusion is that, while both women and men have faced substantial adverse effects under the pandemic and associated lockdowns, the dynamics within the household place women (especially the poor) at greater risk in the long run. Not only do women who have lost their paid jobs face precariousness and dangers, but also women who were unpaid workers in family businesses that have been paralyzed. Furthermore, women may be disproportionately affected not only by the erosion of their own livelihoods, but also by the loss of male jobs and return migration from cities to villages, leading to occupational overcrowding, prolonged domestic work, hunger and even domestic violence. Being less economically resilient, given their restricted work options, women may be displaced by men in some sectors and are likely to have less autonomy and bargaining power within families. These effects could be mitigated to some extent if government and civil society interventions are directed at women, especially with a community approach.

For their part, Bolis et al. (2020) conducted an investigation on five high-income and poor countries in various parts of the world, in order to determine how COVID-19 has affected the unpaid care workload of women and men, and its impact on these individuals’ health, economic security and well-being. The authors find that COVID-19 and the efforts for its containment have led to increases in the unpaid care workload of women and men; however, it has affected them unevenly: although men are dedicating more time to unpaid domestic care work, women continue to do most of it. About half of the women surveyed report that they are now spending more time on unpaid care and domestic work. This has real consequences for the health, economic security and well-being of these women and their families, as almost half of the women surveyed said they felt more anxious, depressed, overworked, isolated or physically ill due to their increased burden of unpaid care and domestic work during the pandemic.

Furthermore, it is highly probable that situations of domestic violence against women have increased in frequency or become more serious, whether these incidents are reported or not. Indeed, the evidence that is emerging indicates in some countries the reporting of violence has increased in the context of economic stress, tension and confinement that configure the COVID-19 crisis, while in other cases, given the difficulty of reporting with the aggressor sharing the same space, or with help services restricted by quarantines, the reporting or requests for help have decreased (United Nations Women, 2020a and 2020b). According to PNUD (2020), in Peru, 23 days after the state of emergency was decreed, more than 8000 calls were answered through Line 100 (the national helpline for cases of domestic violence), which means an average of 360 calls per day, and 43 women were attended. victims of sexual violence, of which more than half were girls.

## Methodological considerations

This paper carries out an extensive descriptive analysis of before and after the start of the Peruvian lockdown for the main labor characteristics of individuals, intersecting them with other characteristics associated with vulnerability to shocks, such as gender, rurality, and family composition. It is important to indicate that in compliance with the Peruvian supreme decree that declared the country in a State of Emergency on March 16, 2020, during the second quarter of 2020 the ENAHO was collected by telephone and with an abridged version of the questionnaire. As of August 2020, face-to-face activities were restarted, and later in September, with the lifting of social isolation, field operations were resumed progressively. From October to December, the survey was carried out throughout the country again through home visits, in-person interviews, and the complete questionnaire (INEI, 2021a).

Although the design of the survey upholds that the necessary care was taken to avoid significant selection biases associated with the information collection method in the context of a pandemic, it is pertinent to explicitly consider the degree of response per quarter, and assess potential distortions. Annex A presents the relevant information on the surveys answered for ENAHO 2020 and their distribution by urban/rural area and by geographic region. The percentage of complete and incomplete surveys (which are the only ones that can be used for any study) went from 71% in the first quarter to 54% in the second quarter, to only 50% in the third quarter, and returned to 76% in the fourth quarter. The category “other”, which includes merged, transitory or demolished dwellings, or a dwellings that are not in use or do not exist, increased significantly in the second and third quarters, most likely for reasons related to the spread of the pandemic. Considering the sampling distribution by urban and rural area, however, and also the distribution by geographic region, no major biases are observed in the making up of the resulting effective quarterly samples (the distributions are very similar). Regarding the households that were excluded from the effective sample, it is apparent that the spatial distribution by geographic region reflects an increase in the exclusion of cases in areas with greater urban movement and agglomeration of people and where the pandemic started and remained strong in the first months of the lockdown (regions such as Metropolitan Lima and the Central Coast), and a corresponding decrease in the exclusion in isolated areas, deemed safe before the risk of contagion (areas such as the Central and South Sierras).

Based on quarterly data contained in the 2020 National Household Survey (ENAHO), the following correspondences are proposed:

- First quarter: January–March 2020. Pre-pandemic period (except for the last fifteen days of March, since the lockdown started on March 16th). Only 10% of the households and 9% of individuals were interviewed after the lockdown began.
- Second quarter: April–June 2020. It coincides with the rigid lockdown period that established mandatory quarantine and physical distancing. Although the lockdown lasted until the end of June, in May a four-phase program was launched to progressively reactivate the economy. The first phase included the mining, construction, and electronic commerce sectors, as well as food delivery services The second phase began in June and incorporated the manufacturing sector related to food, health and transportation goods; commerce of agrarian goods and domestic appliances, and some services such as beauty salons.
- Third quarter: July–September 2020, recovery phase towards the “new normality”. During this quarter, restaurants (but not bars) were allowed to operate at 40% capacity, and domestic travel reinitiated. Commerce in general reopened at 50% capacity, as well as accommodation services, travel agencies and tour operators. Museums also reopened at 50% capacity.
- Fourth quarter: October–December 2020, coincides with the beginning of the fourth phase of the reactivation program. Commerce and stores in general were allowed to function at 60% capacity. Restaurants and related services except bars extended their allowed capacity to 50%. International flights opened up. Libraries, museums, theme parks, archaeological monuments and botanical gardens expanded capacity to 60%.

In order to provide a frame of reference for the best interpretation of the results, some basic characteristics about Peruvian households and their members are presented below. According to annual data ENAHO 2020, approximately one third of Peruvian households and individuals live in rural areas. Within rural areas, 36% of individuals have an indigenous mother tongue, while in urban areas only 10% of individuals do so. At the national level, around 70% of households (80% in rural areas) declare that they are headed by men. Approximately 60% of households are nuclear (made up only of parents and their children), while 28% are extended (that is, they include other relatives in addition to parents and children) and 12% are unipersonal households. In rural areas, the proportion of nuclear households is slightly higher, although only a few percentage points higher. Half of the households are two-parent households, 20% are single-parent households, and 30% are childless households. According to Jaramillo and Ñopo (2020), approximately 84% of two-parent households have children younger than 6 years of age, or dependent members due to illness, disability, or elderly.

One of the most evident gender gaps in Peru is that related to education. While at the national level a third of the principal adults have completed secondary education or have incomplete higher education, this figure hides a distance between men and women of almost 9 percentage points (39% for men versus 30% for women). The greatest contrast to the disadvantage of women is at the level of incomplete primary education: while 24% of women are at this level, 13% of men are. In rural areas, the gap in this educational level is twice as large: 31% for men and 51% for women. The gap reaches an even greater size (25 percentage points) when we distinguish between those who declare that they have an indigenous mother tongue and those who declare that they do not. In this case, among those respondents with an indigenous mother tongue, 26% of men have incomplete primary school or less, while 51% of women are in the same situation.

Informal employment is a structural characteristic of the Peruvian labor market and has shown a growing trend since 2017; seven out of ten Peruvian have informal employment (Gamero and Pérez, 2020). The quality of employment for women within the informal sector is usually lower compared to that of men, since their participation as unpaid family workers is high (INEI, 2021b).

## Results

The main result is that the COVID-19 crisis hit the most vulnerable individuals and households the hardest, exacerbating existing inequalities through multiple channels. In the following pages, results are organized for the principal adults in the household, considered both at the individual and household level. All results are weighted by the corresponding expansion factor. Each of the tables reported allows us to appreciate both the gender gaps in the chosen labor market indicators, as well as the evolution of these gaps throughout 2020 in relation to the development of the COVID-19 crisis. The column corresponding to the first quarter can be considered as a pre-pandemic starting point, since it was only in mid-March that the state of national emergency was declared with a strict quarantine and, as mentioned earlier, few of the observations corresponding to the first quarter were collected after the 16th of March. In addition to the gender gaps, each table also displays the quarterly changes in the indicators chosen for men separately and for women separately, which makes it possible to assess which of the two groups, and under what circumstances, suffered the strongest impacts.

The information at the individual level refers to the “principal adults” of the household, which includes both the heads of the household and their spouses. The term “principal adult” is used to distinguish the one or two adults within the family unit with a decision-making role from any other adults present, and it is also used to generate a broader category of headship or household management, encompassing not only the head (which in its conventional definition is only one individual per household) but also her/his partner, being that in practice it is typically the couple who runs the household.

Since only the principal adults in the household are being considered for the analysis, it is worthwhile to discuss to what extent the broad statistics for these workers—such as labor force participation, hours worked, and the gender gaps—are representative of the national workforce. Annex B compares some key labor market indicators. A first thing to keep in mind is that the group of all the members of the household is younger, as it includes the children of the principal adults. Data not reported on Annex B indicate that the sons and daughters of the household head account for close to 50% of the sample, which brings the average age of the “all household members” group to 32 years of age, compared to 51 years of age for the principal adults group. Well over one third of the all members group are single, and overall they have less education than the household heads. Based on the data presented on Annex B, the principal adults have a higher labor participation and a higher likelihood of being independent workers, in contrast to the all household members group, who have between 5 and 10 percentage points less labor participation and are more likely to be wage workers or unremunerated family workers. Regarding the formality or informality of their activities and the numbers of hours worked, no significant differences are noticeable between the two groups. Probably because of the large portion of the full household members sample that are sons or daughters, the gender gaps are always larger (on personal characteristics as well as on labor market indicators) among the principal adults.

- *Effects of the pandemic on the labor activities of individuals*
  Tables 1, 2, 3 and 4 present the results at the individual level in labor participation, occupational category, informality and number of hours worked per week.
  Table 1 shows the tremendous magnitude that the outbreak of the pandemic had in the employment of Peruvians. Nationally, both principal adults, men and women, suffered a drop of about 26 percentage points with respect to pre-pandemic levels^5^. Considering that the fall was similar, the gender gap in labor participation (around 20 percentage points) remained constant. This makes sense since the declaration of the national lockdown prohibited all economic activities except those considered essential and, according to the estimations by Jaramillo and Ñopo (2020), at the national level a similar percentage of Peruvian men and women were working on sectors deemed non-essential before the pandemic and hence the risk of not being able to work because of the prohibition was similar for both sexes, at least at the aggregate level.^6^
  The most important message in this table, however, does not have to do with the imposition of the quarantine but with the effect caused by its progressive lifting. Indeed, if we consider the column corresponding to the third quarter, we see that male employment levels experienced a much greater recovery (22 percentage points compared to the second quarter) than female employment levels (only 14 percentage points compared to the second quarter). As a consequence, the period of gradual economic reactivation worsened the gender gap in employment, although the gap improves (that is, becomes smaller) in the fourth quarter. A classification of economic sectors according to the risk of losing employment in connection to exposure to the virus allows to explain this situation, as it fits the progressive reactivation of increasingly riskier activities quarter after quarter. Activities in the riskiest sectors were left for last, and women are overrepresented in those sectors, hence it took them longer to rejoin the labor force. Following a methodology developed by ILO, Gamero and Pérez (2020) estimated that 41% of Peruvian employment is in the high-risk sector, and another 8% is in the medium–high risk sector, which meant a high probability that workers in these sectors lost their jobs or were forced to reduce their hours of work, with the corresponding reductions in income. Gamero and Pérez (2020) found that the high risk sector has a high concentration of female employment and youth employment. On average, the proportion of women employed in high-risk sectors is 56%, while the percentage of young people aged 15–29 in these sectors is 30%.
  In other words, an important reason why women were less likely to recover compared to men is because of the pre-existing gendered nature of the labor market, where women are typically employed in more vulnerable kinds of employment compared to men and hence, it was more difficult or took longer for them to go back to work. Yet, another and perhaps more important factor is also at play. As has been noted by a number of studies, a salient aspect of the COVID-19 crisis has involved the closure of schools and daycare centers, implying that children stay at home (OXFAM 2020; Gutiérrez et al. 2020; OIT 2019). This has increased the demands on the time of women, who are usually the primary caretakers in their households. It can be argued, then, that there is also a genuine gendered recovery process whereby, women, irrespective of the kind of the work they are employed in are not able to come back to work, in comparison to men.
  A look at the distinctions by rural and urban area reveals that what happened in urban areas is similar in magnitude and direction to what happened in the national average, while in rural areas the drop in employment was much smaller. This is explained because agricultural activities were considered essential and were not suspended during the quarantine. It is key to note, however, that although the fall in employment in rural areas was less than at the national and urban levels, it was no longer alike for men and women, but much stronger for women. Female job loss in the second quarter was nearly three times greater than male job loss.
  The distinctions in labor participation according to indigenous mother tongue indicate that the gender gaps have grown both for those who declare that they have an indigenous mother tongue and for those who declare that they do not, but much more for the latter. The distinctions in labor participation according to male or female heads of household show that the greatest drops in employment occurred for households headed by women. The gender gaps in labor participation are smaller, however, among these households. It should be kept in mind that although the majority (around 80%) of households headed by women have a female head without a partner, the percentage of those households with a female head who do have a partner is growing. Finally, with regard to distinctions for single versus two-parent households, both the largest falls in labor participation for men and women, as well as the gender gaps were more pronounced among two-parent households.
  Table 2 presents information on the effects of the pandemic on the distribution of occupational categories of household heads and their spouses. The category “unemployed” has been included in order to provide an idea of (i) how occupational categories have changed within the pre-pandemic workforce, and (ii) how much of this change has been because of a movement out of the workforce altogether.
  A marked difference is observed in the patterns of recomposition in occupational categories between men and women as a result of the pandemic (second quarter). While among women there was a large reduction in the presence of independent workers in favor of a small increase of unpaid family workers, among men there was a decrease in the predominance of wage workers with no redistribution among the other occupational categories considered. It is interesting that, with respect to the pre-pandemic levels, both men and women have managed to almost return to the original distribution of their occupational categories, although through different channels. In the second and third quarters the female proportion of unpaid family workers was higher than it was at the beginning of the year, while for men the category that has not fully recovered is that of employers. In terms of gender gaps, in urban areas the greatest change was a decrease of the gap in the category of wage earners, with virtually no change of the gap in unpaid family workers. In rural areas, on the other hand, practically all gender gaps increased, in particular those among unpaid family workers and independent workers, with women shifting from independent work towards unpaid family work. This weakened further the already vulnerable situation of rural women, making them more dependent on the incomes of other family members. Jaramillo and Ñopo (2020) found that there is a strong association between unremunerated family work and the socioeconomic level of the household, with the portion of female unpaid family workers increasing as household income decreases, which implies that women’s vulnerability and lack of economic autonomy were higher among poor households.
  Table 3 presents the effects of the pandemic on the levels of informality of the primary occupation of the principal adults. In general, women are more involved in informal employment than men are. The first column shows that the pre-pandemic gender gap regarding informality was of 11 percentage points. This gap is much smaller in rural areas because both women and men are highly involved in informal work (90% for men and 85% for women). At the national and urban levels, both formal and informal employment decreased in about the same amount among men, while for women the reduction in informal employment was between 3 and 4 times greater than the reduction in formal employment. Clearly, a reason why informality decreased among women in the second quarter, was that they were withdrawing from the workforce entirely, shrinking the share of women in informal work vis a vis formal. Including the out-of-workforce category is particularly important for women since, as some of the studies reviewed in the literature section have confirmed, in the face of the economic distress, women have had limited options to navigate across employment opportunities and are instead, in the majority of cases, forced to withdraw from work altogether.
  The increase of informal employment over the third and fourth quarters, for both women and men but more so for men for the reasons explained above, probably happened first for those independent workers with lesser skills, who engaged in informal work as a way to resume their economic activities and make a living after the decrease or loss of their incomes as a result of the lockdown.
  Table 4 contains information on the number of hours worked per week by principal adult women and men, with breakdowns by urban and rural areas. At the national level, and pre-pandemic levels, just over half of principal men worked between 40 and 60 h per week; among women, only about 32% of them did so (although it is, in any case, the highest proportion of them working across the established ranges of hours). During the second quarter of 2020, it was precisely these ranges of hours that fell the most, particularly among men. The proportion of men working between 40 and 60 hours per week fell by 15 percentage points, while the proportion working more than 60 hours per week fell by 13 percentage points. The falls were less severe for women because to begin with, a smaller proportion of them worked in the higher ranges of hours, but occurred in the ranges of hours mentioned. In the period of economic reactivation, male workers managed to recover almost the same proportion of the distribution of hours in the original ranges to the pre-pandemic levels. Women workers, however, have not been able to return to the same workload as before the start of the crisis. This, together with the findings of the previous table on levels of labor participation, show that for women the recovery or return to normality is happening more slowly, or is not happening fully. Although the ENAHO data do not allow us to analyze the time spent on housework or other activities, the evidence for other countries indicates that confinement at home, with children needing supervision in their schoolwork, with more members at home for whom to cook and cleaning, and also a greater need to care for the elderly in vulnerable situations, are the reason why a significant proportion of women have given up seeking employment, or have reduced their dedication to paid jobs.^7^
  The disaggregations by urban and rural area show that again in rural areas the changes have been less abrupt than in urban areas. In other words, both rural men and women have decreased the number of hours they dedicate to work, but this decrease has not been as strong as that of urban men and women. It should be considered, however, that despite the fact that falls in rural areas have been softer, the largest pre-existing gender gaps (with respect to urban areas) indicate that the situation of women in rural areas actually end up being more unfavorable as a result of the pandemic. For example, for the range of 40–60 hours worked per week, the gender gap in rural areas increased, being larger in the third quarter (29 percentage points)  than in the first quarter (27 percentage points). For the same range of hours, in the urban area the gender gap narrowed, although not by much (17 percentage points in the first quarter versus 15 percentage points in the third quarter).

**Table 1:** Labor participation of household heads and spouses by quarters of year 2020, sex, urban/rural area, mother tongue, household headship and parenthood

	Quarters of 2020															
	First quarter				Second quarter				Third quarter				Fourth quarter			
	Men	Women	Total	Gender gap	Men	Women	Total	Gender gap	Men	Women	Total	Gender gap	Men	Women	Total	Gender gap
Total	**6162**	**6818**	**12,980**		**6291**	**6853**	**13,144**		**6230**	**6738**	**12,968**		**6330**	**6990**	**13,320**	
	**100**	**100**	**100**		**100**	**100**	**100**		**100**	**100**	**100**		**100**	**100**	**100**	
Person worked (%)	81.5	60.9	70.6	20.6	54.8	35.5	44.7	19.3	76.9	49.1	62.4	27.8	84.7	61.9	72.7	22.8
By urban/rural area																
Urban area	78.3	55.6	66.3	22.7	44.8	26.2	35.0	18.6	71.9	42.2	56.3	29.7	81.8	57.0	68.6	24.8
Rural area	92.0	79.2	85.4	12.8	88.4	68.5	78.3	19.9	93.2	73.9	83.5	19.3	94.1	79.7	86.8	14.4
By indigenous mother tongue																
Indigenous mother tongue	84.4	71.3	77.6	13.1	64.1	48.8	56.2	15.3	79.5	59.3	69.3	20.2	86.3	68.2	76.9	18.1
Not indigenous mother tongue	80.5	57.5	68.4	23.0	51.7	31.2	41.0	20.5	75.9	45.7	60.1	30.2	84.0	59.7	71.3	24.3
By household headship																
Male-headed household	81.5	57.8	71	23.7	55.3	33.8	45.9	21.5	77.3	46.0	63.8	31.3	85.0	58.0	73.4	27.0
Female-headed household	80.6	66.7	69.3	13.9	49.4	38.8	40.7	10.6	72.2	54.5	57.9	17.7	81.9	68.0	70.7	13.9
By parenthoold																
Household without children	76.8	58.5	68.3	18.3	48.0	35.8	42.4	12.2	71.3	47.3	60.4	24.0	78.3	61.2	70.1	17.2
Household monoparental	62.6	71.0	69.6	− 8.4	44.2	40.5	41.4	3.7	67.7	58.4	60.0	9.3	73.4	71.2	71.6	2.2
Household biparental	84.2	58.6	71.6	25.6	58.0	33.8	46.1	24.2	79.3	46.5	63.5	32.8	87.7	58.7	73.9	29.0

Source: Instituto Nacional de Estadística e Informática—Encuesta Nacional de Hogares 2020, quarters I, II, III and IV

Weighted results

**Table 2:** Occupational category of household heads and spouses by quarters of year 2020, sex, and urban/rural area

	Quarters of 2020															
	First quarter				Second quarter				Third quarter				Fourth quarter			
	Men	Women	Total	Gender gap	Men	Women	Total	Gender gap	Men	Women	Total	Gender gap	Men	Women	Total	Gender gap
Total	**6162**	**6818**	**12,980**		**6291**	**6853**	**13,144**		**6230**	**6738**	**12,968**		**6330**	**6990**	**13,320**	
	**100**	**100**	**100**		**100**	**100**	**100**		**100**	**100**	**100**		**100**	**100**	**100**	
Employer	6.1	2.5	4.2	3.6	1.8	0.4	1.1	1.4	3.3	0.6	2.0	2.7	5.1	2.1	3.5	3.0
Independent worker	39.4	31.7	35.3	7.7	33.5	14.8	23.7	18.7	39.6	23.3	31.1	16.3	40.4	30.7	35.3	9.6
Wage worker	38.5	18.7	28.1	19.8	25.1	12.8	18.7	12.3	36.4	15.9	25.8	20.5	39.8	19.6	29.2	20.1
Unpaid family worker	2.2	13.4	8.1	− 11.2	1.7	15.1	8.7	− 13.5	2.3	15.0	8.9	− 12.7	2.6	13.8	8.5	− 11.2
Domestic worker	0.2	2.4	1.3	− 2.2	0.2	0.9	0.6	− 0.6	0.2	2.1	1.2	− 1.8	0.2	1.9	1.1	− 1.7
Unemployed	13.6	31.3	22.9	− 17.7	37.7	56.0	47.2	− 18.2	18.2	43.1	31.0	− 24.9	11.9	31.9	22.4	− 20.0
Urban area	**100**	**100**	**100**		**100**	**100**	**100**		**100**	**100**	**100**		**100**	**100**	**100**	
Employer	6.5	2.7	4.5	3.8	1.6	0.4	1.0	1.2	3.5	0.7	2.1	2.8	5.5	2.2	3.7	3.3
Independent worker	30.2	30.0	30.1	0.2	21.3	12.1	16.5	9.2	30.1	21.7	25.7	8.4	31.1	28.6	29.8	2.5
Wage worker	44.5	21.6	32.4	22.9	29.0	14.6	21.4	14.4	41.4	17.7	29.0	23.7	45.8	22.6	33.5	23.2
Unpaid family worker	2.0	5.4	3.8	− 3.3	1.2	4.5	2.9	− 3.4	1.9	5.8	3.9	− 3.9	2.7	5.6	4.3	− 2.9
Domestic worker	0.1	2.7	1.6	− 2.6	0.2	1.1	0.7	− 0.8	0.3	2.5	1.4	− 2.2	0.3	2.4	1.4	− 2.1
Unemployed	16.7	37.6	27.7	− 20.9	46.7	67.3	57.5	− 20.6	22.8	51.6	37.9	− 28.8	14.6	38.6	27.3	− 24.0
Rural area	**100**	**100**	**100**		**100**	**100**	**100**		**100**	**100**	**100**		**100**	**100**	**100**	
Employer	4.7	1.6	3.1	3.0	2.3	0.5	1.4	1.8	2.7	0.7	1.7	2.0	3.9	1.5	2.7	2.4
Independent worker	69.8	37.6	53.2	32.2	74.2	23.9	48.5	50.3	70.6	29.0	49.8	41.6	70.7	38.3	54.3	32.4
Wage worker	18.8	9.1	13.8	9.8	12.1	6.7	9.4	5.5	20.1	9.2	14.7	10.9	20.0	8.8	14.3	11.2
Unpaid family worker	2.9	41.5	22.8	− 38.6	3.5	52.9	28.7	− 49.4	3.5	48.4	25.9	− 45.0	2.1	43.1	22.9	− 41.0
Domestic worker	0.2	0.7	0.4	− 0.5	0.3	0.1	0.2	0.2	0.1	0.4	0.2	− 0.3	0.1	0.4	0.3	− 0.3
Unemployed	3.6	9.5	6.7	− 5.9	7.6	15.9	11.8	− 8.2	3.0	12.3	7.7	− 9.2	3.2	7.9	5.6	− 4.7

Source: Instituto Nacional de Estadística e Informática—Encuesta Nacional de Hogares 2020, quarters I, II, III and IV

Weighted results

**Table 3:** Informal employment (for primary job) of household heads and spouses by quarters of year 2020 and urban/rural area

	Quarters of 2020															
	First quarter				Second quarter				Third quarter				Fourth quarter			
	Men	Women	Total	Gender gap	Men	Women	Total	Gender gap	Men	Women	Total	Gender gap	Men	Women	Total	Gender gap
Total	**6108**	**6650**	**12,758**		**6232**	**6630**	**12,862**		**6180**	**6572**	**12,752**		**6274**	**6809**	**13,083**	
	**100**	**100**	**100**		**100**	**100**	**100**		**100**	**100**	**100**		**100**	**100**	**100**	
Informal employment	57.1	51.4	54.1	5.7	43.5	30.9	37.0	12.6	57.8	42.2	49.8	15.6	61.1	51.2	56.0	9.9
Formal employment	28.8	15.3	21.8	13.5	18.5	9.8	14.0	8.7	23.8	12.0	17.7	11.8	26.7	14.9	20.5	11.8
Unemployed	14.1	33.3	24.1	− 19.3	38.0	59.3	49.0	− 21.3	18.4	45.8	32.5	− 27.4	12.2	33.9	23.5	− 21.6
Urban area	**100**	**100**	**100**		**100**	**100**	**100**		**100**	**100**	**100**		**100**	**100**	**100**	
Informal employment	47.2	42.4	44.7	4.8	30.3	19.7	24.8	10.5	47.4	32.3	39.5	15.1	52.1	42.1	46.9	10.0
Formal employment	35.7	18.8	26.8	16.9	22.8	11.9	17.0	11.0	29.6	14.6	21.8	15.0	32.9	18.1	25.0	14.8
Unemployed	17.1	38.8	28.5	− 21.7	46.9	68.4	58.2	− 21.5	23.0	53.1	38.7	− 30.1	15.0	39.8	28.1	− 24.8
Rural area	**100**	**100**	**100**		**100**	**100**	**100**		**100**	**100**	**100**		**100**	**100**	**100**	
Informal employment	89.9	84.5	87.2	5.3	88.2	74.5	81.5	13.7	91.8	80.1	86.1	11.7	90.4	86.2	88.3	4.2
Formal employment	6.1	2.4	4.2	3.8	3.8	1.8	2.8	2.0	4.7	2.0	3.4	2.7	6.3	2.7	4.6	3.6
Unemployed	4.0	13.1	8.6	− 9.1	8.0	23.7	15.7	− 15.7	3.5	17.9	10.5	− 14.3	3.3	11.1	7.1	− 7.8

Source: Instituto Nacional de Estadística e Informática—Encuesta Nacional de Hogares 2020, quarters I, II, III and IV

Weighted results

**Table 4:** Total weekly hours worked by household heads and spouses by quarters of year 2020 and urban/ rural area

	Quarters of 2020															
	First quarter				Second quarter				Third quarter				Fourth quarter			
	Men	Women	Total	Gender gap	Men	Women	Total	Gender gap	Men	Women	Total	Gender gap	Men	Women	Total	Gender gap
**Total**	**5495**	**4835**	**10,330**		**4546**	**3502**	**8048**		**5425**	**4078**	**9503**		**5748**	**4904**	**10,652**	
	**100**	**100**	**100**		**100**	**100**	**100**		**100**	**100**	**100**		**100**	**100**	**100**	
Less than 10 h/week	6.0	11.5	8.6	− 5.5	14.1	16.5	15.2	− 2.4	6.6	13.1	9.4	− 6.5	4.1	9.5	6.6	− 5.4
Between 10–19 h/week	5.0	12.5	8.5	− 7.5	11.0	17.8	13.9	− 6.8	8.2	15.6	11.4	− 7.4	5.6	12.6	8.8	− 7.0
Between 20–39 h/week	19.0	28.4	23.3	− 9.4	32.3	36.7	34.2	− 4.4	26.0	34.7	29.8	− 8.7	21.4	28.4	24.6	− 7.1
Between 40–60 h/week	51.6	31.9	42.4	19.7	36.8	25.3	31.8	11.5	49.3	29.7	40.9	19.6	52.3	35.4	44.6	16.9
More than 60 h/week	18.4	15.7	17.2	2.7	5.8	3.7	4.9	2.1	9.9	6.9	8.6	3.0	16.6	14.0	15.4	2.6
Urban area	**100**	**100**	**100**		**100**	**100**	**100**		**100**	**100**	**100**		**100**	**100**	**100**	
Less 10 than hours/week	6.7	12.7	9.5	− 6.0	18.5	20.5	19.3	− 2.0	7.9	14.8	10.7	− 6.9	4.3	10.1	6.8	− 5.8
Between 10–19 h/week	4.4	11.5	7.6	− 7.1	11.1	14.0	12.2	− 2.9	8.8	13.8	10.8	− 5.0	5.6	10.8	8.0	− 5.2
Between 20–39 h/week	16.6	23.4	19.7	− 6.8	29.6	31.5	30.4	− 1.9	23.9	29.8	26.3	− 5.9	19.4	23.9	21.4	− 4.5
Between 40–60 h/week	51.4	34.4	43.6	17.0	34.2	29.0	32.2	5.2	48.1	32.9	41.9	15.2	52.4	38.8	46.3	13.6
More than 60 h/week	20.9	18.1	19.6	2.8	6.6	5.0	5.9	1.6	11.3	8.7	10.3	2.6	18.3	16.4	17.5	1.9
Rural area	**100**	**100**	**100**		**100**	**100**	**100**		**100**	**100**	**100**		**100**	**100**	**100**	
Less than 10 h/week	3.8	8.5	6.1	− 4.7	5.7	11.1	8.4	− 5.5	3.1	9.6	6.2	− 6.5	3.4	8.1	5.7	− 4.7
Between 10–19 h/week	6.6	15.1	10.8	− 8.5	10.8	23.0	16.8	− 12.2	6.6	19.4	12.7	− 12.8	5.4	16.9	11.1	− 11.5
Between 20–39 h/week	25.8	40.5	33.1	− 14.7	37.5	44.0	40.6	− 6.5	31.7	44.6	37.8	− 12.9	27.1	39.4	33.1	− 12.3
Between 40–60 h/week	52.5	25.9	39.3	26.6	41.5	20.0	31.0	21.5	52.4	23.3	38.6	29.1	52.2	27.4	40.0	24.8
More than 60 h/week	11.3	10.0	10.7	1.3	4.5	1.9	3.2	2.6	6.2	3.1	4.7	3.1	11.9	8.2	10.1	3.7

Source: Instituto Nacional de Estadística e Informática—Encuesta Nacional de Hogares 2020, quarters I, II, III and IV

**Graph 1:**
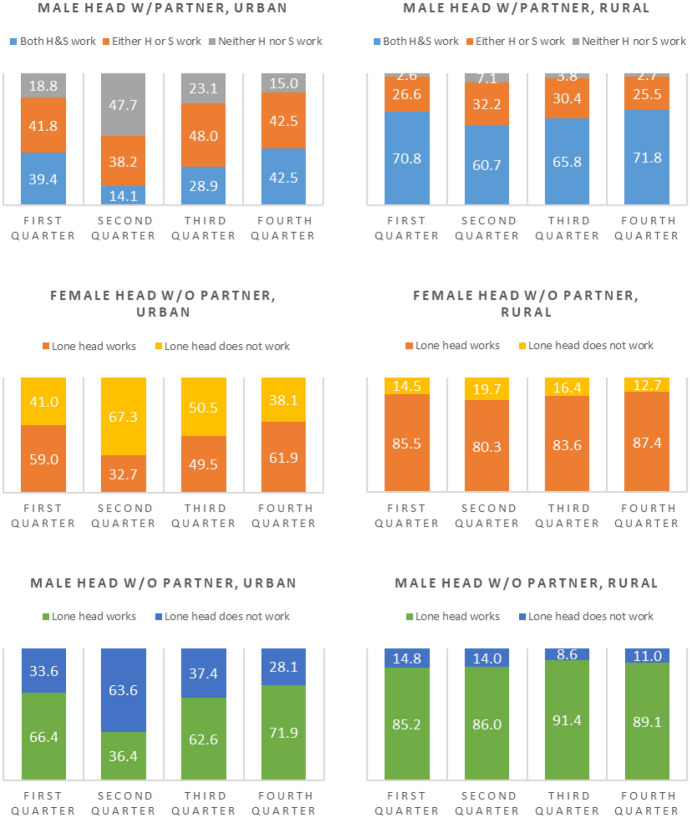
Joint labor participation of household heads and spouses by quarters of year 2020, household headship, and urban/rural area. *H* Head, *S*  Spouse; Source: Instituto Nacional de Estadística e Informática—Encuesta Nacional de Hogares 2020, quarters I, II, III and IV. Weighted results

**Graph 2:**
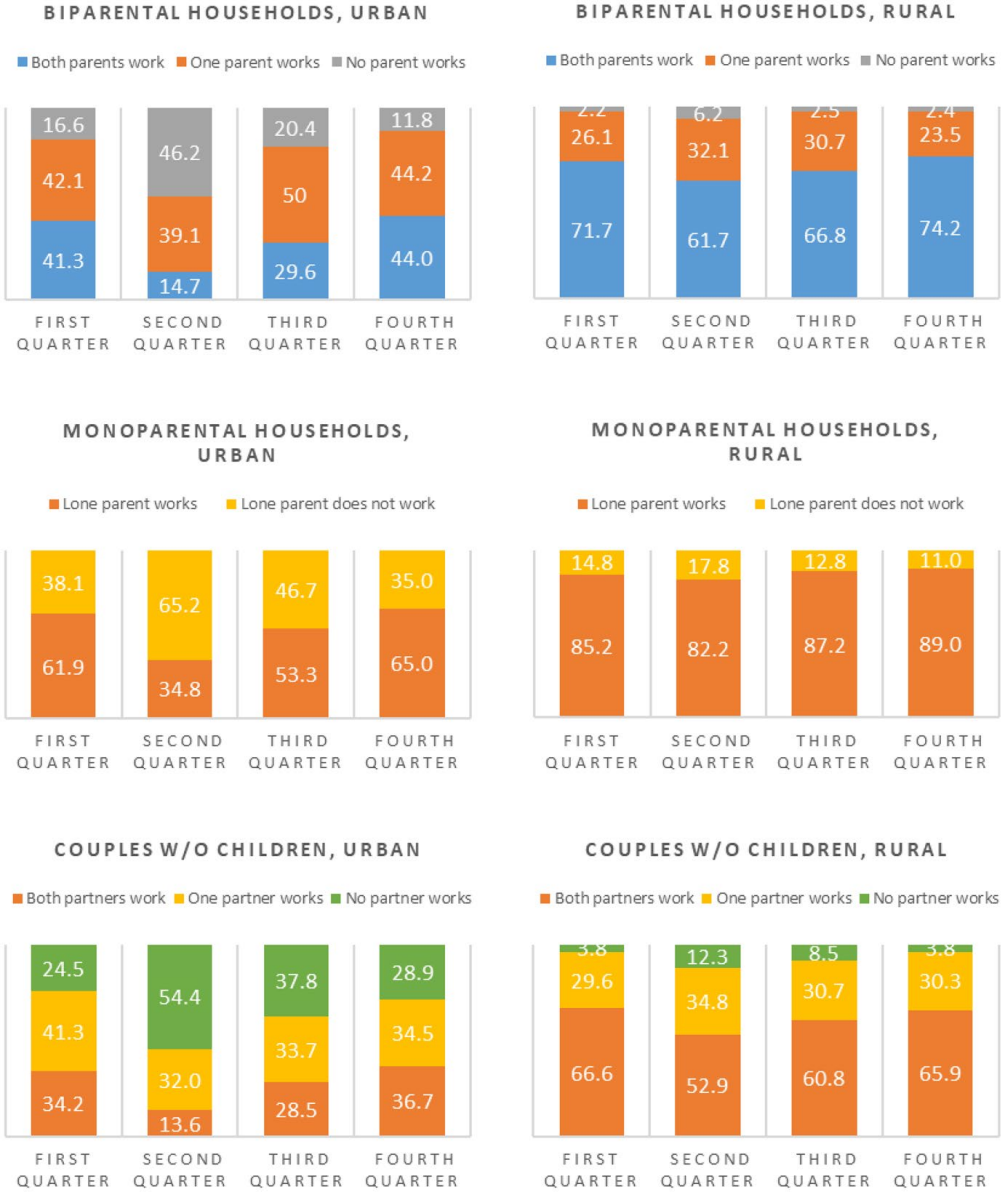
Joint labor participation of household heads and spouses by quarters of year 2020, parenthood, and urban/ rural area. Source: Instituto Nacional de Estadística e Informática—Encuesta Nacional de Hogares 2020, quarters I, II, III and IV. Weighted results
  Graph1 distinguishes between principal adults according to male or female heads of household and according to whether the heads are in a situation of having a partner or not, and by urban and rural area. As can be seen, in urban areas, among households headed by men and by women without a partner, the first and second quarter appear quite similar regarding whether the lone household head was working or not. The big difference occurs in the reactivation period (third quarter), in which a much lower percentage of households headed by a lone female register participation in the labor market. Considering that 71% of these households have children (compared to only 27% of households headed by a lone male; data nor presented in the graph), the situation of potential vulnerability in which urban households headed by a woman without a partner is clear. The dynamics unleashed by the pandemic, which as mentioned before have to do with the increase in domestic work and care work, with no help from a partner, make it more difficult for urban lone women who have lost their jobs to recover them. Additionally, urban single female headed households are more vulnerable as a result of the economic sectors they are engaged in. While urban lone female heads work primarily (83%) in commerce and services activities, a much smaller proportion of urban lone male heads (55%) do so, while another 31% of them engage in manufacturing and construction. Although in the third quarter commerce and services were being reactivated, caring for their children probably stopped these women from rejoining the labor force.
  Graph 2 presents the results of the combined strategies of the principal adults with disaggregations based on single parenting, biparenting, or the absence of children in the household. In accordance with what was found in the tables of results at the individual level for labor participation, in monoparental urban households, the loss of employment between the first and second quarters was of the order of around 25 percentage points. Among rural monoparental households there are no appreciable differences across quarters. A more interesting finding refers to those urban households headed by a man or woman with a partner, and with the presence of children, that is, urban two-parent households. As can be seen in the second quarter column, the changes with respect to the pre-pandemic situation occur between the category in which both parents work and the category in which neither parent works. That is, there does not seem to be an intermediate situation in which one of the members of the couple worked and then stops working, but the other member continues to work. The most common situation seems to be the one in which both members of the couple lost their jobs. This is connected with the fact that in the first quarter, 20% of urban biparental household had both principal adults working in the sector or commerce and services. In another 19% of the cases, only one member of the couple was working and it did so in commerce and services activities. It is understandable, then, that when that sector was shutdown in the second quarter, a large portion of these urban biparental households became households in which neither principal adult was working at all. In the third quarter, however, in the period of economic reactivation, there were situations in which one of the members started working while another member remained unemployed. The highest proportion of cases is grouped around situations in which both members of the couple recovered their employment, however, which reinforces the possibility that both members of the couple actually work in similar economic sectors.
  The differentiations according to urban and rural areas once again confirm that the strongest changes were registered in urban areas, which includes not only falls but also recoveries.

Weighted results
- *Effects of the pandemic on the labor activities of households*
  Graph 1 and 2 present the results for the joint participation of household heads and spouses in the labor market. For the classification of households, specifications of different configurations were used: women and men alone or with a partner; within households with a partner, joint labor force participation between the partners (for example, one partner lost her/his job, and the other partner did not; both partners lost their job, etc.).

## Conclusions and recommendations

The COVID-19 pandemic has become one of the most serious challenges the world has faced in recent times. In addition to the loss of human life, a general decline in economic activity has been unleashed that is affecting and will continue to affect people's well-being for many years. Several of the actions that governments took to control contagions, such as quarantines and mandatory social distancing, reduced and in many cases paralyzed production and consumption activities, with the consequent fall of markets and closure of companies, and the loss of employment for millions of people. This paper estimated the differentiated impacts of the COVID-19 crisis in Peru using the nationally representative surveys to gain knowledge of the situation from a proactive and inclusive perspective.

The main conclusion is that the pre-existing conditions and inequalities, expressed both in the urban/rural spatial distribution, and in the socioeconomic vulnerabilities contained in the female/male household headship, as well as the presence of children and having or not having a partner, configured that those most disadvantaged individuals and households faced greater impacts on the main labor market indicators. As explained in the results tables, in some cases the impact of the pandemic itself (visualized in the columns corresponding to the second quarter of 2020) was even between men and women and therefore did not worsen the existing gender gaps, but rather that the disadvantages of the most vulnerable were identified in the third quarter, when evaluating the recovery of indicators as a result of the gradual “return to normality”. Rural households, households headed by women (most of which are headed by women without a partner), and single-parent households are the ones that are having the most problems in recovering to pre-pandemic levels for the selected labor indicators.

The realization that the COVID-19 crisis hits the most vulnerable individuals and households the most requires that the policies that the government implements in the coming months, or that it designs for the next couple of years, prioritize those that were already in a disadvantageous position before the pandemic. They should take into account that women, particularly those who are heads of households without a partner, who live in rural areas, are the group that has borne the greatest burden in dealing with the economic and social disruptions that accompany public health crises. As mentioned at the beginning of the document, total or partial confinement demands that women dedicate more time to housework and caring for others, which hinders their reintegration into the labor market. Specific cash transfers for women with the aforementioned vulnerabilities could be a short-term policy option.

As the national economy gradually reactivates, it is crucial to understand that many of the inequities observed in the labor market, and that have been exacerbated by the pandemic, have to do with traditional gender roles. In a broader time horizon, it is important that the State promotes more equitable gender roles, where men and women share work within the home with greater equality. In general, all gender equality policies should be reinforced.

As in all countries in the world, the COVID-19 crisis has profoundly affected all aspects of daily life in Peru, from public and private health care, to job security, organization within the home, mobility, among many others. Understanding how the government measures taken so far have heterogeneously affected different population groups is essential to design and implement policies that really help to recover the economy and well-being of Peruvians at least to pre-pandemic levels. Not differentiating sufficiently between different segments of individuals and households could be hiding important evidence for populations at higher risk.

## Data Availability

At request. Raw data available at http://iinei.inei.gob.pe/microdatos/.

## Annex

See Annex A and B.

**Annex A:** Final results of households surveyed for enaho 2020 by quarters of year 2020, urban/rural area, and geographical region

	Quarters of 2020			
	First quarter	Second quarter	Third quarter	Fourth quarter
Full sample (*N*)	**11,412**	**14,854**	**16,040**	**10,990**
Total	**100**	**100**	**100**	**100**
Survey complete	58.4	51.7	46.3	65.9
Survey incomplete	12.2	2.5	3.7	10.4
Survey rejected	5.1	2.6	2.7	4.4
Absent / vacant housing	9.2	5.8	5.6	8.2
Other	15.1	37.4	41.7	11.1
Included in sample (complete + incomplete)	**8063**	**8055**	**8014**	**8377**
	**100**	**100**	**100**	**100**
Urban/rural distribution				
Urban area	78.4	79.7	78.9	77.9
Rural area	21.6	20.3	21.1	22.1
Distribution by geographic region				
Costa norte	13.5	13.3	12.8	13.2
Costa centro	6.9	6.9	7.3	6.8
Costa sur	2.6	2.7	3.0	3.1
Sierra norte	5.0	5.4	5.0	5.5
Sierra centro	13.1	11.6	12.1	12.4
Sierra sur	15.6	15.6	15.3	16.0
Selva	11.7	11.8	12.2	12.3
Lima Metropolitana	31.6	32.7	32.3	30.7
Excluded from sample (all other cases)	**3349**	**6799**	**8026**	**2613**
	**100**	**100**	**100**	**100**
Urban/rural distribution				
Urban area	84.0	88.1	82.0	85.2
Rural area	16.0	11.9	18.0	14.8
Distribution by geographic region				
Costa norte	9.1	10.4	10.1	9.9
Costa centro	3.5	5.3	7.7	4.0
Costa sur	2.5	3.0	3.0	3.3
Sierra norte	3.2	2.2	3.7	3.4
Sierra centro	11.6	7.3	9.0	9.8
Sierra sur	21.8	14.3	19.1	20.0
Selva	8.7	11.3	10.7	8.8
Lima Metropolitana	39.6	46.2	36.5	40.8

Source: Instituto Nacional de Estadística e Informática—Encuesta Nacional de Hogares 2020, quarters I, II, III and IV

Weighted results

**Annex B:** Differences between principal adults and all households members on selected labor market indicators

	Principal adults				All household members			
	Men	Women	Total	Gender gap	Men	Women	Total	Gender gap
Labor participation								
Total	**26,821**	**29,334**	**56,155**		**45,417**	**45,812**	**91,229**	
At the national level	76.2	52.7	63.9	23.4	65.2	47.0	56.1	18.1
Urban area	71.5	47.1	58.5	24.5	60.6	42.4	51.3	18.2
Rural area	92.2	75.9	84.2	16.4	83.1	68.5	76.3	14.6
Occupational category								
Total	**22,959**	**18,527**	**41,486**		**34,131**	**26,516**	**60,647**	
%	**100**	**100**	**100**		**100**	**100**	**100**	
Employer	4.7	2.0	3.5	2.6	3.5	1.6	2.6	1.9
Independent worker	48.6	43.2	46.2	5.5	37.6	33.8	35.9	3.9
Wage worker	43.7	27.1	36.3	16.6	47.6	33.3	41.4	14.3
Unpaid family worker	2.7	24.1	12.1	− 21.4	10.7	27.3	18.0	− 16.6
Domestic worker	0.3	3.6	1.8	− 3.2	0.6	4.0	2.1	− 3.4
Informal employment								
Total	**22,609**	**17,206**	**39,815**		**32,751**	**23,864**	**56,615**	
At the national level	72.1	79.7	75.4	− 7.6	73.9	77.3	75.3	− 3.3
Urban area	64.0	73.2	68.0	− 9.2	66.7	70.8	68.4	− 4.1
Rural area	94.5	97.3	95.7	− 2.8	95.2	97.2	96.0	− 2.0
Hours worked per week								
Total	**22,959**	**18,527**	**41,486**		**34,131**	**26,516**	**60,647**	
%	**100**	**100**	**100**		**100**	**100**	**100**	
Less than 10 h/week	7.3	11.8	9.3	− 4.6	7.8	12.3	9.9	− 4.4
Between 10–19 h/week	6.9	14.6	10.4	− 7.7	8.8	15.7	11.8	− 6.9
Between 20–39 h/week	24.0	32.2	27.7	− 8.2	24.5	30.3	27.0	− 5.8
Between 40–60 h/week	48.8	30.2	40.4	18.6	47.1	31.7	40.3	15.4
More than 60 h/week	13.0	11.2	12.2	1.8	11.8	10.0	11.0	1.8

Source: Instituto Nacional de Estadística e Informática—Encuesta Nacional de Hogares 2020, annualized versión

Weighted results

## Footnotes

1 Updated information available at https://covid19.minsa.gob.pe/sala_situacional.asp.

2 Updated information available at https://coronavirus.jhu.edu/data/mortality.

3 The Oxford COVID-19 Government Response Tracker (OxCGRT) systematically collects information (currently from more than 180 countries) on several different common policy actions that governments have taken to respond to the pandemic, differentiating across various degrees of rigidity. The data is also used to inform a set of four indices, including the stringency index (Hale et al., 2020). This index considers whether the country has carried out closure of schools and universities; closure of workplaces and businesses belonging to non-essential economic activities; cancellation of public events; prohibition of social gatherings; closure of public transportation; requirements to stay at home, and mandatory quarantine for the entire population; restrictions to domestic travel; and restrictions to international travel.

4 The Banco Central de Reserva (2020) estimated a 12% drop in the national GDP in 2020, which puts Peru among one of the most affected countries in the world. For Latin America and the Caribbean, the consensus of estimates gauges an average annual fall of between 3 and 4% in the region's gross product (Lustig and Tommasi, 2020).

5 This coincides with the official estimates, which although calculated with respect to the second quarter of 2019, place the fall of the economic activity rate at 28 percentage points for women and 25 percentage points for men (INEI 2020a– 2020e).

6 Using data from ENAHO 2018, Jaramillo and Ñopo (2020) estimated that 44% of Peruvian women and 42% of Peruvian men worked in non-essential activities. In urban areas the figure reached 51% for both women and men.

7 On average, Peruvian men dedicate approximately 16 h per week to domestic work, while women dedicate almost 40 h per week (INEI, 2021b). There are no estimations of how the pandemic has affected these averages.
